# Protective effect of inhalation of hydrogen gas on radiation-induced dermatitis and skin injury in rats

**DOI:** 10.1093/jrr/rru067

**Published:** 2014-07-17

**Authors:** Sadahiro Watanabe, Masanori Fujita, Masayuki Ishihara, Shoichi Tachibana, Yoritsuna Yamamoto, Tatsumi Kaji, Toshio Kawauchi, Yasuhiro Kanatani

**Affiliations:** 1 Department of Radiology, National Defense Medical College, 3–2 Namiki, Tokorozawa, Saitama 359–8513, Japan; 2 Division of Environmental Medicine, National Defense Medical College Research Institute, 3–2 Namiki, Tokorozawa, Saitama 359–8513, Japan; 3 Second Division, Aeromedical Laboratory, Japan Air Self-Defense Force, 1-2-10 Sakae, Tachikawa, Tokyo 190–8585, Japan; 4 Division of Biomedical Engineering, National Defense Medical College Research Institute, 3–2 Namiki, Tokorozawa, Saitama 359–8513, Japan; 5 Department of Health Crisis Management, National Institute of Public Health, 2-3-6 Minami, Wako, Saitama 351–0197, Japan

**Keywords:** radioprotection, hydrogen, radiodermatitis, healing-impaired wound, antioxidant

## Abstract

The effect of inhalation of hydrogen-containing gas (1.3% hydrogen + 20.8% oxygen + 77.9% nitrogen) (HCG) on radiation-induced dermatitis and on the healing of healing-impaired skin wounds in rats was examined using a rat model of radiation-induced skin injury. An X-ray dose of 20 Gy was irradiated onto the lower part of the back through two holes in a lead shield. Irradiation was performed before or after inhalation of HCG for 2 h. Inhalation of HCG significantly reduced the severity of radiodermatitis and accelerated healing-impaired wound repair. Staining with terminal deoxynucleotidyl transferase-mediated dUTP nick-end labeling (TUNEL) and 8-hydroxy-2^′^-deoxyguanosine (8-OHdG) showed that the proportion of apoptotic keratinocytes and the level of staining in the X-irradiated skin of rats that pre-inhaled HCG were significantly lower than that of rats which did not pre-inhale HCG. Cutaneous full-thickness wounds were then created in the X-irradiated area to examine the time-course of wound healing. X-irradiation significantly increased the time required for wound healing, but the inhalation of HCG prior to the irradiation significantly decreased the delay in wound healing compared with the control and post-inhalation of HCG groups. Therefore, radiation-induced skin injury can potentially be alleviated by the pre-inhalation of HCG.

## INTRODUCTION

Radiotherapy is a widely accepted treatment for various types of cancers. However, acute injurious effects of radiation on the skin, such as erythema, epilation, desquamation, hyperpigmentation and erosion, collectively referred to radiodermatitis, are common side-effects, depending on the irradiation dose [1, 2]. In addition, chronic radiation-induced skin ulcers are often observed in the region surrounding radiodermatitis [3]. These ulcers are characterized by poor healing and a high relapse rate, and are commonly intractable. These symptoms are not only the limiting factors during radiotherapy, but are also public health concerns, forcing the interruption or termination of the therapeutic course. Therefore, a treatment is required to reduce those side-effects without reducing the anti-tumor effects. Recently it was reported that consumption of hydrogen-rich water reduces the biological reaction to radiation-induced oxidative stress without compromising the anti-tumor effects [4].

X-irradiation interacts with water molecules in biological systems and produces various reactive oxygen species (ROS) that can cause cell and tissue damage, together with induced excess cellular apoptosis [5]. ROS such as O_2_^−^ and H_2_O_2_ are easily detoxified by antioxidant defense enzymes, whereas cytotoxic ROS, such as the hydroxyl radical (·oh), cannot be detoxified by these enzymes. During the process of radiotherapy, the detrimental effects of ionizing radiation on biological tissues such as skin are mostly mediated by cytotoxic ROS such as the hydroxyl radical, inducing excess cellular apoptosis [6, 7].

The sulfhydryl amifostine (WR-2721), which is the only radioprotectant registered for use in humans, shows good radioprotectant effects [8]. However, many side-effects limit its clinical use, such as hypertension, nausea and vomiting [9]. Some natural antioxidants, such as vitamins and flavonoids, have fewer toxic effects but also provide lower radioprotection [10]. An ideal radioprotectant would be effective and cause mild side-effects. To date a radioprotectant which can fulfill these criteria is not available.

Ohsawa *et al*. [11] have demonstrated that molecular hydrogen (H_2_) can selectively reduce cytotoxic ROS. In addition, H_2_-therapy also could be a useful way to protect human skin fibroblasts from oxidative damage [12], as it has been shown to have a positive effect on excess cellular apoptosis and acute radiodermatitis [6]. Furthermore, H_2_-inhalation was reported to mitigate cerebral [10], myocardial [13] and hepatic [14] radiation injury in animal models. Buchholz *et al*. [15] also reported that H_2_-inhalation ameliorates oxidative stress in transplantation-induced intestinal graft injury. Thus, H_2_ may be a novel antioxidant with significant potential to be used in safe and effective medical applications without causing any known side-effects.

A beneficial effect of intraperitoneal pre-injections of Prostaglandin E1 has been demonstrated for radiation-induced healing-impaired skin injury [5]. However, the intraperitoneal injection of a high dose of Prostaglandin E1 for radiation-induced healing-impaired skin injury may have side-effects [5]. In particular, a high dose of Prostaglandin E1 may induce severe changes in blood pressure or bleeding via vasodilation or platelet aggregation. Furthermore, although an advantage of intraperitoneal injection of hydrogen-rich saline has been demonstrated for radiation-induced dermatitis [6], the inhalation of hydrogen-containing gas (HCG) may be an easier and safer pre-treatment technique to prevent dermatitis than intraperitoneal injection of hydrogen-rich saline. To the best of our knowledge, no previous studies have examined the effects of inhalation of HCG to stimulate the healing of radiation-induced skin injury by reducing cytotoxic ROS and preventing radiation-induced apoptosis of skin cells. The purpose of this study was to evaluate the effects of inhalation of HCG on alleviating radiodermatitis and on stimulating the healing of skin injuries.

## MATERIALS AND METHODS

### Rats and feeding conditions

All animal experiments were carried out according to the protocol approved by the Animal Experimentation Committee at the National Defense Medical College (Tokorozawa, Saitama, Japan). Ambient temperature was kept at 25 ± 2°C during the experiments. Male Sprague–Dawley rats weighing ∼350 g (10 weeks old) were purchased from Japan SLC (Hamamatsu, Shizuoka, Japan) and given *ad libitum* access to food and water. Animals were lightly sedated in an ether-filled anesthesia box. After sedation, intraperitoneal anesthesia was performed using a 27-gauge injection needle. The anesthetic agent was a 5 mg/ml solution of pentobarbital sodium (Somnopentyl^®^, 64.8 mg/ml; Kyoritsu Seiyaku, Tokyo, Japan) in physiological saline, injected at an initial dose of 50 mg/kg body weight.

### HCG treatment of rats

Before or after X-irradiation, rats were treated with HCG (hydrogen: 1.3%, oxygen: 20.8%; nitrogen: 77.9%) (Saisan Inc., Saitama, Japan) fed into an acrylic box (250 × 250 × 300 mm; Shinano Manufacture Inc., Tokyo, Japan) at a flow rate of 2 l/min. The hydrogen in the box was monitored using a gas detector (TH-D4A; KOMYO RIKAGAKU KOGYO K.K., Kanagawa, Japan). Pre-inhalation rats were treated with HCG for 2 h just before X-irradiation, and post-inhalation rats were treated with HCG for 2 h just after X-irradiation.

### Radiation apparatus

Before the irradiation of dorsal skin, the hair was shaved using electric clippers. The distance between the radiation source and the rat on the turntable was 35 cm, and an MBR-1505R2 X-ray irradiation apparatus (Hitachi Medical Corporation, Tokyo, Japan) was used. The radiation factors were 150 kV, 5 mA, with 0.5 mm Al added filtration. The dose rate was 1 Gy/min. The animals were protected with 3 mm-thick lead shielding containing 2 holes (diameter: 2.3 cm), thus allowing controlled irradiation of target patches of dorsal skin [5].

### Effect of inhalation of HCG on early radiation-induced dermatitis and healing-impaired wounds

Early radiodermatitis was measured 4 weeks after X-irradiation. The degree of early cutaneous toxicity was evaluated 3, 7, 14, 21 and 28 d after X-irradiation, according to the skin score criteria [6]. Three observers scored the blinded groups of rats using the skin score criteria (Table 1) [6].

**Table 1.** The criteria for radiation-induced acute dermatitis of rat dorsal skin

Score	Observation
0.0	No change
0.5	Faint erythema
1.0	Bright erythema
1.5	<50% dry desquamation
2.0	>50% dry desquamation, mild edema
2.5	<50% moist desquamation, moderate edema
3.0	>50% moist desquamation, moderate edema
3.5	Erosion, strong edema
4.0	Ulceration

Skin reactions were evaluated macroscopically from Grade 0 to 4 in nine steps, according to clinical criteria of cancer radiotherapy. The observation period was 4 weeks after irradiation.

The areas X-irradiated with 20 Gy through the two holes (2.3-cm diameter) were marked, then cutaneous full-thickness wounds were made with an 8-mm biopsy punch. The wounds made in the four groups (pre-, post- and non-inhalation of HCG, and control (non X-irradiation)) were evaluated on Days 3, 5, 7, 10, 14 and 20 after wound formation. Each rat was kept separately without a wound dressing. Several pictures of the irradiated skin area and wound were taken with a digital camera (DiMAGE X50; Konica Minolta, Tokyo, Japan) and a ruler on each day. The size (mm^2^) of each wound was measured from the digital pictures using software for measurements (Adobe Photoshop CS5, Adobe System Inc., San Jose, USA). The wound-healing rate was calculated by the following formula: healing rate (%) = (original wound area – unepithelialized area)/original wound area × 100 [5]. Healing refers to a combination of wound constriction and epithelization, not including crust formation and granulation.

### Immunohistochemistry

Excised skin tissues were fixed in 4% paraformaldehyde solution at 4°C for 12 h, embedded in paraffin, and cut into 3.5-μm-wide sections 5, 24 and 72 h after irradiation, and 7 d after irradiation. Sections were made perpendicular to the anteroposterior axis and perpendicular to the surface of the skin. Each section was stained with hematoxylin and eosin.

Cellular apoptosis assessed by DNA fragmentation was examined using a MEBSTAIN apoptosis TUNEL Kit II (MBL Co. Ltd, Nagano, Japan). Skin sections were deparaffinized and exposed to proteinase K (20 μg/ml) for 15 min at room temperature to unmask the fragment DNA 3′-OH ends. Samples were washed with distilled H_2_O and incubated in 0.3% hydrogen peroxide for 15 min to suppress endogenous peroxidase activity. After two rinses in phosphate-buffered saline (PBS), sections were sequentially incubated in equilibration buffer at room temperature for 10 s. After tapping off excess liquid from the samples, terminal deoxynucleotidyl transferase enzyme was applied, and the samples were incubated at 37°C for 1 h. Stop buffer was applied to the samples at room temperature for 15 min to terminate the 3′-OH labeling reaction, and peroxidase-conjugated streptavidin (Dako, Glostrup, Denmark) was applied at room temperature for 30 min. After washing the samples in PBS, 3-3′-diaminobenzine-4HCl (DAB) (Dako, Glostrup, Denmark) was added, and the samples were stained for 5 min. The sections were counterstained with Mayer's hematoxylin (Merck, Darmstadt, Germany) to study tissue morphology.

For staining with 8-OHdG, skin sections were deparaffinized, washed with distilled H_2_O, and incubated in 0.3% hydrogen peroxide for 15 min to suppress endogenous peroxidase activity. After washing with PBS, blocking solution was applied to the samples at room temperature for 10 min to adsorb non-specific proteins. The washed samples were treated with primary antibody (anti-8-OHdG mouse monoclonal antibody, Japan Institute for the Control of Aging, Shizuoka, Japan) for 12 h at 4°C. The washed samples were treated with secondary antibody (anti-mouse peroxidase-conjugated second antibody, Nichirei Biosciences, Tokyo, Japan) for 30 min at room temperature. After washing the samples, DAB was added and the samples were stained for 5 min. The sections were counterstained with Mayer's hematoxylin to study tissue morphology.

The total number of epidermal keratinocyte cells (EKCs) was counted in four adjacent fields using a light microscope (40 × objective and 10 × photo eyepiece). Each field represented a total length of ∼300 μm per skin sample. The mean number of total EKCs and apoptotic or 8-OHdG-positive EKCs in each skin sample harvested from the four groups (pre-, post- and no-inhalation of HCG and control (no X-irradiation)) were evaluated by the proportions calculated by the following formula: apoptotic (or 8-OHdG positive) cell count (%) = apoptotic (or 8-OHdG positive) EKC counts/total EKC counts × 100.

The values of 8-OHdG and malondialdehyde (MDA) in serum prepared from venous blood were measured using an 8-OHdG and MDA ELISA kit (Nikken Zairu Inc., Shizuoka, Japan) according to the manufacturer's instructions.

### Statistical analysis

Results are given as mean ± SD. Comparisons of mean number of apoptotic cells between the control and experimental groups were determined by Student's *t*-test. Otherwise, a Kruskal–Wallis (KW) test was used for comparisons among groups, and Dunn's test was used for multiple comparisons. Statistical analyses were conducted using StatMate III for Windows (ATMS Co., Ltd, Tokyo, Japan). *P*-values of < 0.05 were considered significant.

## RESULTS

### Effect of inhalation of HCG for radiodermatitis

**Fig. 1.**
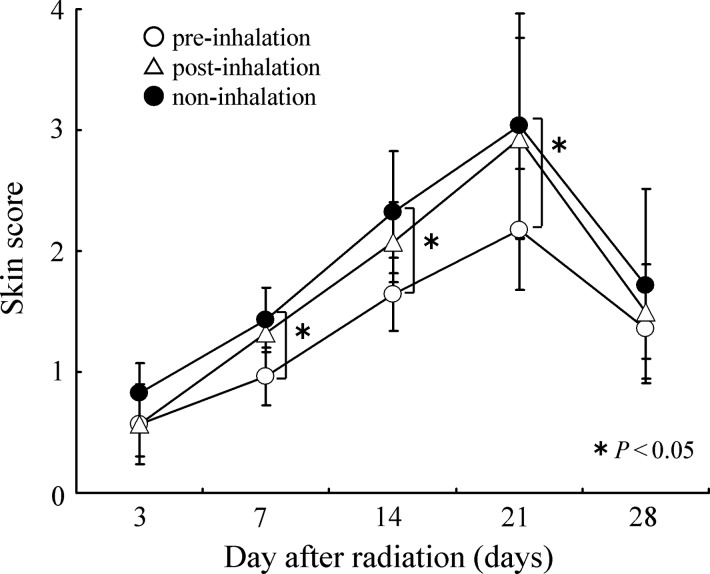
The chronological change in the skin score for acute dermatitis after 20-Gy irradiation. In each group (*n* = 14), scores peaked at 3 weeks and then tended to decrease. A significant radioprotection effect was observed in the pre-inhalation of HCG group compared with the post- and non-inhalation group on Days 7, 14 and 21.

In non- and post-inhalation rats that received a 20-Gy X-irradiation dose, erythema with epilation was observed 7 d after irradiation, and desquamation and edema were observed ∼14 d after irradiation. The most extensive skin damage and inflammation caused by X-irradiation was observed 21 d after irradiation (Table 1 and Fig. 1). In contrast, pre-inhalation rats suffered significantly less damage compared with the non- and post-inhalation rats (Table 1 and Fig. 1).

### Effects of inhalation of HCG for irradiation-induced wound healing

**Fig. 2.**
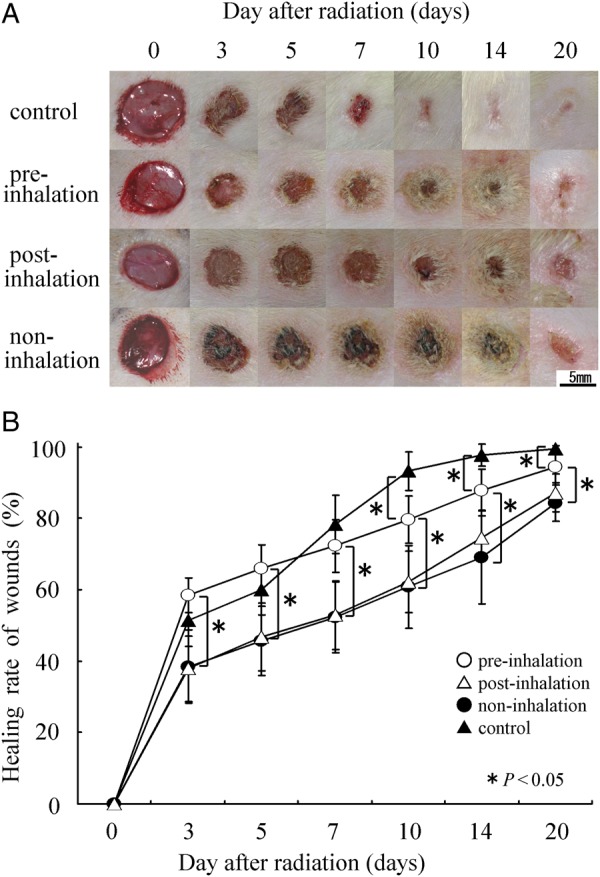
Healing rates of a cutaneous full-thickness wound with or without 20-Gy irradiation. The irradiation groups inhaled HCG pre- or post-irradiation, or did not inhale HCG. The control group did not receive irradiation or inhale HCG. Wound-healing rates were measured in each group (*n* = 14) on Days 3, 5, 7, 10, 14 and 20 post-irradiation. (**A**) Each wound size (mm^2^) was measured by the picture of each day. Scale bars = 5 mm. The wound healing rate (%) = (original wound area – unepithelialized area)/original wound area × 100. (**B**) Signficant improvements in wound healing were observed in the pre-inhalation group compared with the post- and non-inhalation groups. There were no significant differences between the post- and non-irradiation groups.

For the non-inhalation of HCG rats, comparison of the irradiated and non-irradiated groups showed significantly delayed wound healing in the irradiated group. Over half the rats in the irradiated group showed incomplete healing after 2 weeks, with complete wound healing taking 20 d. Significant differences in wound-healing rate were also observed in the different HCG inhalation groups following 20-Gy irradiation (Fig. 2A and B): the pre-inhalation group showed significantly better healing rates from 3 d to 20 d post-irradiation (3, 5, 7, 10, 14 and 20 days) (*P* < 0.05) compared with the non- and post-inhalation group. Also, there was no significant difference in the healing rates at all timepoints between the post- and non-inhalation group (*P* > 0.05).

### TUNEL and 8-OHdG staining of irradiated skin

**Fig. 3.**
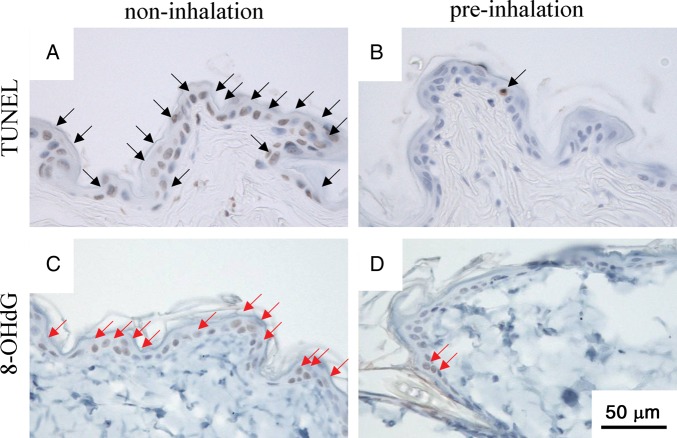
TUNEL and 8-OHdG staining of skin sections 24 h after 20-Gy irradiation. Apoptotic cells were evaluated by TUNEL-staining (**A**, **B**), and the hyperoxidation state of the nucleic acids were evaluated by 8-OHdG staining (**C**, **D**). The number of epidermal cells was counted in four adjacent fields of each skin section using a light microscope (×40 objective and ×10 photo eyepiece; Scale bars = 50 μm). There were significant decreases in the number of apoptotic cells (black arrow) and 8-OHdG-positive cells (red arrow) in the pre-inhalation group (B, D) compared with the non-inhalation group (A, C).

**Fig. 4.**
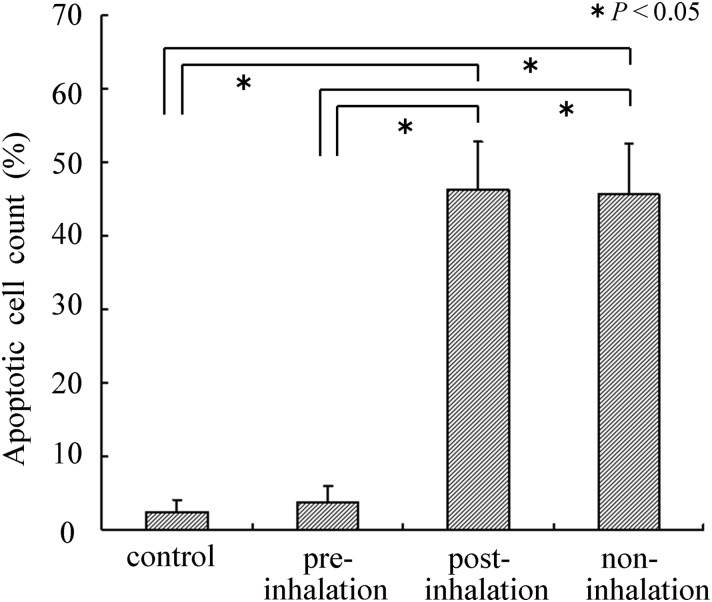
TUNEL-staining of skin sections 24 h after 20-Gy irradiation was performed to measure the proportion of apoptotic cells. TUNEL indicates DNA fragmentation. A significant decrease in the number of apoptotic cells was observed in the pre-inhalation group compared with the post- and non-inhalation groups. The number of apoptotic cells observed in the pre-inhalation group was equal to that in the control group (non-irradiation).

TUNEL staining showed a large number of apoptotic epidermal keratinocyte cells (EKCs) in the non- and post-inhalation HCG group from 5 h to 7 d post-irradiation. The proportion of apoptotic cells per total EKCs in the epidermal tissue of the non- and post-inhalation group was significantly higher compared with the proportion in the pre-inhalation group on Day 1 (Fig. 3 and Fig. 4), Day 3 and Day 7 (data not shown).

**Fig. 5.**
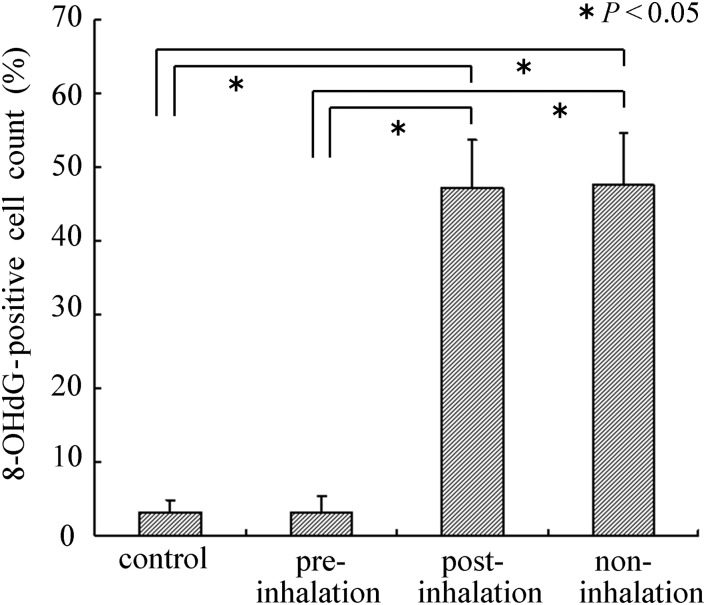
8-OHdG immunohistochemistry staining of skin sections 24 h after 20-Gy irradiation was performed to measure the proportion of 8-OHdG-positive cells. 8-OHdG indicates the hyperoxidation state of the nucleic acids, allowing the anti-oxidative effect to be evalutated. A significant decrease in the number of 8-OHdG-positive cells was observed in the pre-inhalation group compared with the post- and non-inhalation groups. The decrease in the number of 8-OHdG-positive cells observed in the pre-inhalation group was equal to that of the control group (non-irradiation).

Similarly, 8-OHdG staining showed a large number of stained EKCs in the epidermal tissues of the non- and post-inhalation HCG group from 5 h to 7 d post-irradiation. We expect that the reduction in the number of 8-OHdG stained cells is related to the direct scavenging of hydroxyl radicals by hydrogen gas inhaled before irradiation. The proportion of stained cells per total EKCs in the epidermal tissue of the non-inhalation group was significantly higher compared with the proportion in the pre-inhalation group on Day 1 (Fig. 3 and Fig. 5), Day 3 and Day 7 (data not shown).

### Changes in the concentrations of serum MDA and 8-OHdG

The concentration of serum MDA and 8-OHdG in the pre-, post- and non-inhalation groups were measured 5 h after irradiation, when the maximal increase in serum MDA and 8-OHdG was observed. The concentration of serum MDA was 188 ± 47, 184 ± 49, 162 ± 42 and 142 ± 59 (nM) in the non-, post- and pre-inhalation HCG groups, and in the control (non-irradiation), respectively. The concentration of serum 8-OHdG was 8.1 ± 1.4, 7.7 ± 2.1, 7.2 ± 1.9 and 7.0 ± 1.6 (ng/ml) in the non-, post- and pre-inhalation HCG groups, and in the control (non-irradiation), respectively. Although there was a slight increase in the serum concentration of MDA and 8-OHdG in the non- and post-inhalation HCG groups compared with the pre-inhalation group, these were not statistically significant.

## DISCUSSION

It has previously been demonstrated that hydrogen gas selectively reduces cytotoxic ROS such as the hydroxyl radical [11, 16]. The hydroxyl radical is the most reactive ROS generated in cells. Hydroxyl radicals can easily react with cellular molecules, including DNA, proteins and lipids, to exert a strong cytotoxic effect [17, 18]. Since most of the ionizing radiation-induced damage is caused by hydroxyl radicals, we speculated that the radioprotective effect of hydrogen gas may result from its ROS scavenging effect. On the other hand, cellular apoptosis is critical to many physiological processes, including embryogenesis, immune cell maturation and response, tissue homeostasis, and in the cellular response to injury [19]. In pathological states, excessive apoptosis may contribute to organ and tissue injury. Thus, selective preservation of cell modulation during apoptotic processes could affect the treatment of many diseases. Previous studies have indicated that apoptosis plays an important role in radiation-induced injuries on epidermal keratinocytes (EKCs) [5, 20], thymus lymphocytes [19, 21], hepatocytes [14] and bone marrow cells [22]. The previous study showed that X-irradiation induced apoptosis of EKCs in rat skin *in vivo* and inhibited the proliferation of these cells *in vitro* [5]. The present study demonstrated that pre-inhalation of hydrogen-containing gas (HCG) alleviates radiodermatitis (Fig. 1) and stimulates the healing of radiation-induced skin injury (Fig. 2) by reducing cytotoxic ROS and preventing the radiation-induced apoptosis of EKCs.

X-irradiation-induced radiodermatitis includes erythema, epilation, depigmentation and erosion. Our results (Fig. 1) showed that pre-inhalation of HCG significantly alleviated acute radiodermatitis compared with post- and non-inhalation of HCG. When cutaneous full-thickness wounds were formed in the irradiated areas in order to examine the time-course of wound healing, healing was found to be significantly delayed because of skin injuries induced by irradiation. Pre-inhalation of HCG resulted in significantly shorter delays in wound healing compared with post- and non-inhalation of HCG (Fig. 2). These results suggest that HCG inhalation prior to X-irradiation is effective for alleviating radiation damage to the skin, as well as for promoting wound healing.

**Fig. 6.**
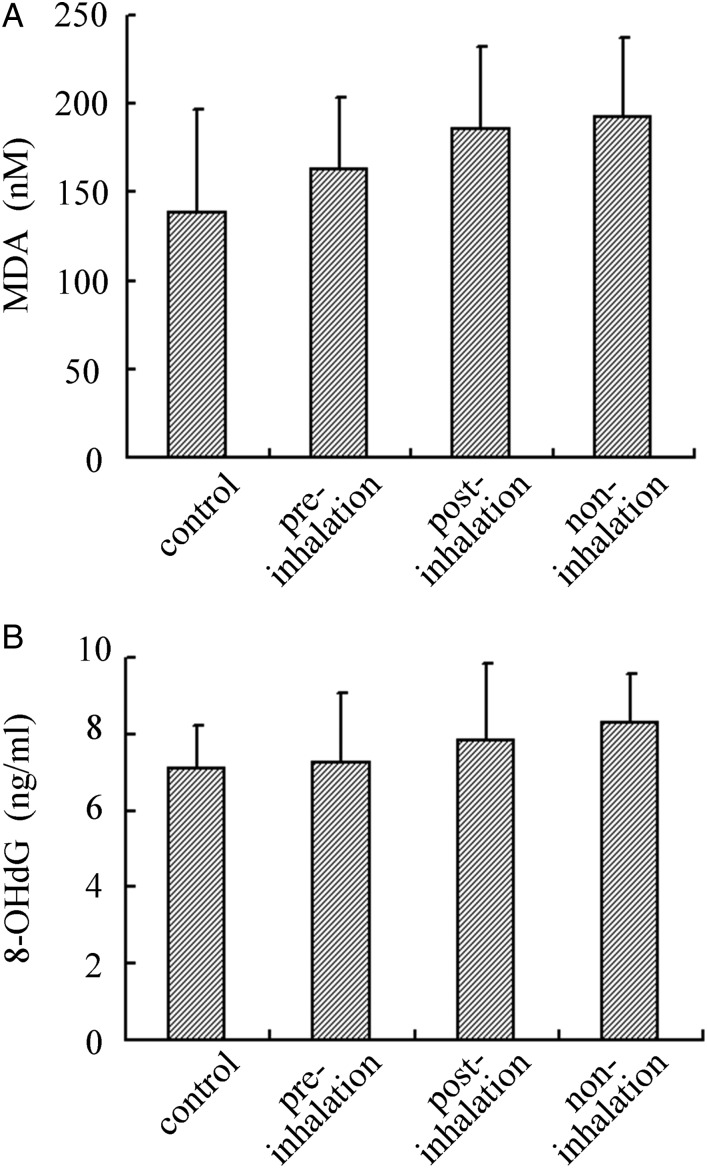
The concentration of serum oxidation stress marker was measured 5 h after 20-Gy irradiation. In each group (*n* = 8), there was no significant deficiency in serum MDA(**A**) and 8-OHdG(**B**), although a slight decrease in serum MDA and 8-OHdG in the pre-inhalation group was observed compared with the post- and non-inhalation groups.

The radioprotective, antiapoptotic and antioxidant mechanisms by which pre-inhalation of HCG exerts its effects remain to be studied *in vitro* and *in vivo.* This *in vivo* study showed that pre-inhalation of HCG significantly reduced the severity of X-radiation-induced oxidative injury and the apoptosis responses in X-radiated skin areas, as evidenced by the levels of 8-OH-dG and TUNEL staining (Figs. 3–5). Thus, it is believed that the inhibitory effect for the apoptosis of EKCs may be caused by inactivation of the cytotoxic ROS generated by X-irradiation, which could ameliorate cell apoptosis and skin damage. On the other hand, there were no significant differences in MDA and 8-OH-dG levels in blood 5 h after X-irradiation among the pre-, post- and non-inhalation of HCG groups (Fig. 6). Based on our present study, only local radiation-induced skin damage can be alleviated by administering HCG before irradiation.

Mild cases of skin damage caused by irradiation can be healed using ointments, but severe cases cannot be healed using conservative therapy. In those cases, some type of surgical procedure may be required. For example, when performing a skin graft, if the graft bed is irradiated, the formation of granulations and blood vessels are typically decreased, and skin graft survival rate also decreases. Moreover, a skin flap that is damaged by radiation may result in a poor flap survival rate, i.e. if the area surrounding a lesion is irradiated, treatment methods will be less effective because of the effects of radiation. Therefore, a simple method that can reduce such damage as much as possible is required. The inhalation of HCG agrees with many requirements of an ideal radioprotectant, such as efficacy, broad spectrum, acceptable administration and non-toxicity [23].

In conclusion, we showed here that pre-inhalation of HCG (1.3% hydrogen + 20.8% oxygen + 77.9% nitrogen) for 2 h prior to X-irradiation produced no known toxic side-effects and effectively reduced the severity of dermatitis and accelerated skin tissue recovery. Thus, the inhalation of HCG may be an easier and safer pre-treatment to prevent the dermatitis. The pre-inhalation of HCG may provide a new clinical therapy in the treatment of radiodermatitis and oxidative damage caused by radiation treatment.

## FUNDING

This work was funded by the National Defense Medical College. Funding to pay the Open Access publication charges for this article was provided by National Defense Medical College, Japan.
